# Humidity Sensing Properties of Paper Substrates and Their Passivation with ZnO Nanoparticles for Sensor Applications

**DOI:** 10.3390/s17030516

**Published:** 2017-03-04

**Authors:** Georgios Niarchos, Georges Dubourg, Georgios Afroudakis, Markos Georgopoulos, Vasiliki Tsouti, Eleni Makarona, Vesna Crnojevic-Bengin, Christos Tsamis

**Affiliations:** 1 Nano and Microelectronics Group, BioSense Institute, 1 Zorana Djindijca, 21000 Novi Sad, Serbia; georgesdubourg@uns.ac.rs (G.D.); bengin@uns.ac.rs (V.C.-B.); 2 Institute of Nanoscience and Nanotechnology, NCSR “Demokritos”, Patriarhou Gregoriou & Neapoleos, 15310 Aghia Paraskevi, Greece; george.afroudakis@gmail.com (G.A.); m.georgopoulos@imperial.ac.uk (M.G.); v.tsouti@inn.demokritos.gr (V.T.); e.makarona@inn.demokritos.gr (E.M.); c.tsamis@inn.demokritos.gr (C.T.)

**Keywords:** humidity passivation, paper substrate, ZnO nanoparticles, disposable sensors

## Abstract

In this paper, we investigated the effect of humidity on paper substrates and propose a simple and low-cost method for their passivation using ZnO nanoparticles. To this end, we built paper-based microdevices based on an interdigitated electrode (IDE) configuration by means of a mask-less laser patterning method on simple commercial printing papers. Initial resistive measurements indicate that a paper substrate with a porous surface can be used as a cost-effective, sensitive and disposable humidity sensor in the 20% to 70% relative humidity (RH) range. Successive spin-coated layers of ZnO nanoparticles then, control the effect of humidity. Using this approach, the sensors become passive to relative humidity changes, paving the way to the development of ZnO-based gas sensors on paper substrates insensitive to humidity.

## 1. Introduction

Cellulose fiber-based paper has been used for long time in missives, flyers, books or newspapers, because it is both chemically and mechanically stable under atmospheric conditions and absorbs ink readily. In the present-day, paper can be also used as substrate for the fabrication of electronic devices such as antennas, sensors, photovoltaics or energy storage devices [1,2,3,4]. Compared to other substrates like silicon or glass, paper as a substrate offers an important number of advantages: the raw materials required for its manufacturing process are very abundant organic materials, it is inexpensive, lightweight, recyclable, disposable and environmentally benign [5]. In addition, recent advancements in printed electronics using electrically functional inks and traditional printing technologies such as flexo, screen, offset and gravure, allow the fabrication of paper-based electronic devices in a simple, large-scale and low-cost manner [6,7,8].

Paper is a good candidate for gas-sensing applications thanks to its inherent porous structure and rough surface, which provides a large surface area compared to flat glass or a nonporous plastic substrate [9]. Several kinds of gas sensors have already been presented in the literature like ethanol, hydrogen sulfide or nitrogen dioxide sensors [10,11,12] and are based on metal oxides [13], nanoparticles [14] or polymers [15,16].

Paper is also the perfect candidate for low-cost and disposable humidity sensors due to its capacity to absorb water vapor. However, only a few concrete examples of humidity sensors using paper substrate as the sensing element can be found in the literature. For instance, a fully printed chip-less radio frequency identification sensor for humidity monitoring applications was reported in [17]. Another example presents a capacitive-type humidity sensor using silver interdigitated electrodes printed on paper substrate. This work investigates the effect of water molecule absorption on recycled paper capacitance and conductance [18]. The ability of a paper to absorb moisture largely depends on its manufacturing process, since this process affects the morphology of cellulose fibers on its surface. Therefore, we need to further investigate paper manufacturing methods.

Although paper can be a promising candidate for humidity sensing, its use for other gas sensors can be restricted by the same effect, affecting the gas sensor’s selectivity and influencing their performances. Many studies have focused on devising methods for improving the surface hydrophobicity of paper, such as laser ablation to modify the surface morphology and/or change the surface energy [19] and plasma-induced polymerization to create hydrophobic polymer chains on the paper’s surface to make it water repellent [20]. Other interesting examples include the possibility to improve surface hydrophobicity of paper by using a coating of organic or inorganic nanoparticles [21,22]. Moreover, an interesting technique is proposed in [23], which describes a coating process aimed at reducing the effect of humidity on paper-based electronics. Even though this process is effective, it requires a specific type of paper, takes longer, and incurs additional costs.

In this context, we propose a simple, effective and low-cost approach to study and reduce the effect of humidity on the electrical properties of simple printing papers with different morphology. As a first step, we fabricated paper-based microdevices by laser ablating metal layers deposited on the surface of the paper substrates and characterized them in detail under known relative humidity levels at room temperature. This enabled us to explore the role of surface morphology in the ability of a particular paper to act as a humidity sensor.

As a second step, we examined a time- and cost-efficient methodology—inspired by Nature’s approach to exploit nanoparticle formations for functionality enhancement [24]—as a potential route for passivating paper substrates with respect to humidity. The development of the proposed methodology was partly based on a previous work that employed sol-gel solutions with suspended ZnO nanoparticles in an attempt to control the wetting properties of wood surfaces [25]. ZnO nanostructures [13,26] (nanoparticles, nanowires, nanoflakes, etc.) are exceptionally interesting due to their great potential in many practical applications such as energy harvesting [27], sensors [28] and solar cells [29].In our case, we thoroughly investigated how ZnO nanoparticles affect sensor operation, in an attempt to pave the way for disposable and selective sensors on paper substrates with no impact from humidity.

## 2. Materials and Methods

### 2.1. Fabrication of Paper-Based Devices

**Figure 1:**
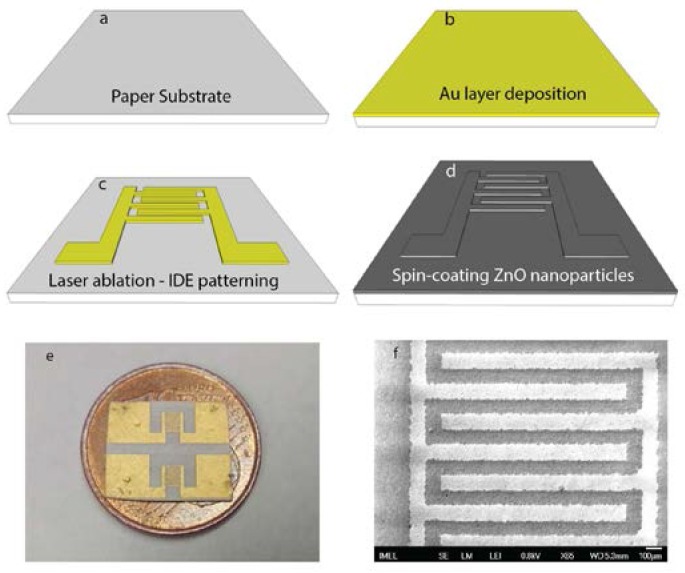
Microdevice fabrication process. (**a**) Paper substrate, (**b**) Deposition of Au layer, (**c**) Laser micromachining of the interdigitated electrodes, (**d**) Spin-coating the ZnO-nanoparticle layers, (**e**) Photograph of the finished fabricated devices on paper (2 devices on a coin of 1 cent), (**f**) Scanning Electron Microscope (SEM) image of the resulting nanotextured ZnO film.

Even though the most popular paper used in sensor technology is the Whatman no.1 chromatography paper (Sigma-Aldrich Chemie Gmbh, Taufkirchen, Germany), this work focuses on two other paper substrates: a plain printing paper of a 80 gm^−2^ basis weight and a glossy, photographic quality paper of a 200 gm^−2^ basis weight. Interdigitated electrodes (IDEs) were directly patterned on them using an inexpensive and fast fabrication process, which does not require the high-cost semiconductor manufacturing equipment normally used for silicon microfabrication. The process sequence is schematically illustrated in Figure 1.

The papers were cut into 3 × 2 cm^2^ rectangular pieces (Figure 1a) that were loaded onto a sputtering deposition system and a thin layer of Au/Cr (100 nm/10 nm) was directly deposited on them (Figure 1b) without any pre-treatment, showing good adhesion properties. The resulting layer was directly patterned by laser ablation using a short pulse laser (Nd:YAG-1064 nm, Rofin-Sinar Laser Gmbh, Hamburg, Germany), in order to define the IDEs design (Figure 1c).

The laser ablation process was optimized by modification of the current values at a constant raster speed and selection of appropriate frequencies. The maximum available frequency of 65 kHz and a low raster speed of 80 mm/s were chosen to obtain well-defined electrodes and selective ablation of the metal layer without destroying the paper as shown in Figure 1e.

### 2.2. Preparation of ZnO Nanoparticles

Synthesis of one-dimensional ZnO-based nanostructures has been achieved with various techniques such as electrodeposition [30], hydrothermal method [31], sol-gel method [32], pyrolysis [33] and the commercial method [34]. The sol-gel method, which is the most widely used, offers good homogeneity, low processing temperatures and large-area coating, is low-cost and allows control of the solution composition.

In our work, sol-gel solutions were prepared by dissolving zinc acetate dihydrate (Zn(CH_3_COO)_2_.2H_2_O, Merck Millipore, Darmstadt, Germany) into analytical grade ethanol (C_2_H_6_O, Carlo Erba Reagents S.A.S., Val de Ruil, France) at a concentration of 40 mM [25]. The resulting suspended ZnO nanoparticles were deposited on the paper substrates using successive spin-coatings (Figure 1d), the number of which was ranged from one to ten for the 40 mM solution. After each coating step, the samples were annealed at 100 °C for 10 min on a hotplate in order for ZnO nanoparticles to bind together and form a film and to remove any traces of the solvent left inside the paper. This temperature was selected in order to avoid deformation of both the electrodes and the paper itself. Figure 1f shows a photograph of the fabricated microdevice on the paper along with a magnified Scanning Electron Microscope (SEM) image of the nanotextured ZnO film on the electrodes.

**Figure 2:**
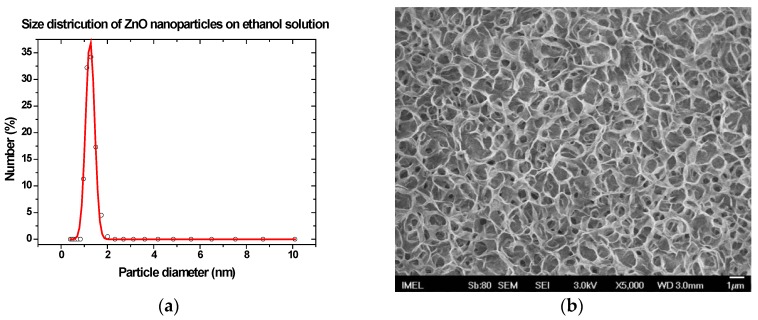
(**a**) Size distribution of suspended ZnO nanoparticles in sol-gel solution and (**b**) SEM image of a patterned with IDEs Si wafer coated with 15 layers of ZnO nanoparticles from a 40 mM sol-gel.

Prior to the deposition of the nanoparticles, their size was measured using a Zetasizer Nano ZS (Malvern Instruments Ltd., Worcestershire, UK) their size distribution shown in Figure 2a. The sol-gel process involves hydrolysis, condensation and polymerization of monomers, growth of particles and agglomeration. After nucleation and growth, the average particle size may change by aging, leading to unsystematic agglomeration, formation of large monodisperse nanoparticles, compact or porous polycrystalline micrometer-size particles, spheres or faceted particles.

In order to examine whether the successive coating with the ZnO sol-gel results in a uniform film, a patterned silicon wafer with IDE electrodes was deposited with ZnO nanoparticles under the same conditions (40 mM ZnO sol-gel with thermal annealing between successive coatings). The SEM image of the resulting film (Figure 2b) shows that a porous film forms, even on a smooth and solid surface like Si. This indicates that for the selected spin coating conditions film uniformity and structural morphology on the papers may also depend on surface roughness.

### 2.3. Measurement Setup

A custom-designed experimental setup was developed in order to perform the measurements in a controlled environment. The setup consists of a sealed Teflon chamber, inside which the sample is carefully placed, along with the sensing tip of a portable hydrometer, in order to measure the changes in the RH in real-time. Mass-flow controllers and flow meters from Brooks Instruments (Hatfield, PA, USA) were used to determine the actual flow rates and concentration of gases. Nitrogen was used as a carrier gas for the water vapors. The gas was driven inside a sealed glass bottle containing water (bubbler), which caused the formation of bubbles and the resulting vapors were then guided to the Teflon chamber where the device was located. Responses to relative humidity changes were recorded by measuring the drop in resistance across the IDEs, using an amperometer (Keithley, Tektronix, Inc., Beaverton, OR, USA) driven by a custom-designed Labview-based interface. All measurements were performed in room temperature (T = 25 °C).

## 3. Results and Discussion

### 3.1. Structural Characteristics of Uncoated Devices

The effect of humidity on paper largely depends on its surface morphology and composition, which in turn depend on the manufacturing process. A structural characterization of both types of paper is thus required in order to understand paper behavior under controlled humidity levels.

**Figure 3:**
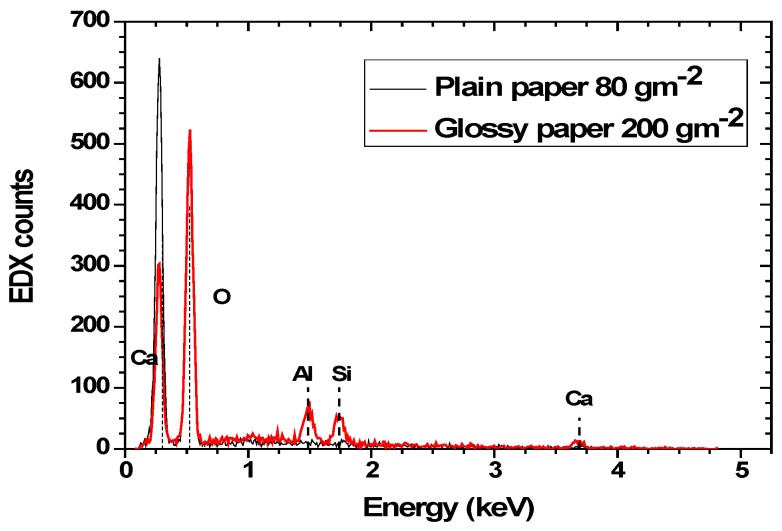
EDX spectra of the 80 gm^−2^ plain paper and the 200 gm^−2^ glossy paper.

Energy-dispersive X-ray spectroscopy (EDX) was used in order to analyze the composition of the various papers. Figure 3 is the EDX spectrum of both the plain and glossy paper where the presence of calcium and oxygen can clearly be identified. The existence of these elements is due to the calcium carbonate (CaCO_3_) introduced into the pulp during paper manufacturing in order to fill the pores of the cellulose matrix and improve paper quality, especially light scattering, ink absorbance and surface smoothness. Similar results were also obtained for the glossy paper. However, in this case, in addition to calcium and oxygen, silicon, as well as aluminum peaks were identified.

**Figure 4:**
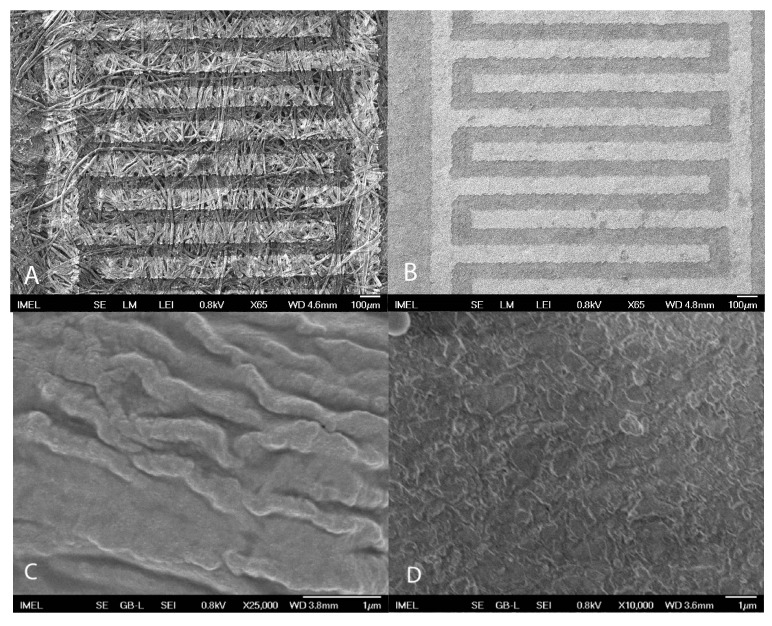
SEM images of the surface of the (**A**) 80 gm^−2^ plain paper and (**B**) 200 gm^−2^ glossy paper, (**C**) and (**D**) are higher magnifications of each film respectively.

Although the exact manufacturing conditions of the paper substrates used is not known, the presence of Si and Al is not unusual since it can be attributed to the specifics of the manufacturing processes for improving paper quality and characteristics. Dry-strength additives, or dry-strengthening agents, are chemicals that improve paper strength under normal conditions. These improve the paper’s compression strength, bursting strength, tensile breaking strength, and delamination resistance. Typical chemicals used include cationic starch and polyacrylamide (PAM) derivatives. These substances work by binding fibers, often with the aid of aluminum ions in paper sheet. In addition, Si is used during paper fabrication, a fact that could explain the EDX signal. SEM characterization of both paper substrates with patterned IDE was performed in order to determine the differences in morphology, as shown in Figure 4.

The cellulose fibers that form the paper structure can be clearly identified in the case of devices on the 80 gm^−2^ plain paper indicating a rough and porous surface morphology (Figure 4A), while the 200 gm^−2^ glossy paper exhibits a smoother surface and appears more compact (Figure 4B).

### 3.2. Humidity Characteristics of Uncoated Devices

Depending on its surface structure, paper has the inherent ability to absorb water molecules resulting to a change in its electrical properties. Thus, we must consider the effect of humidity on plain and glossy papers.

**Figure 5:**
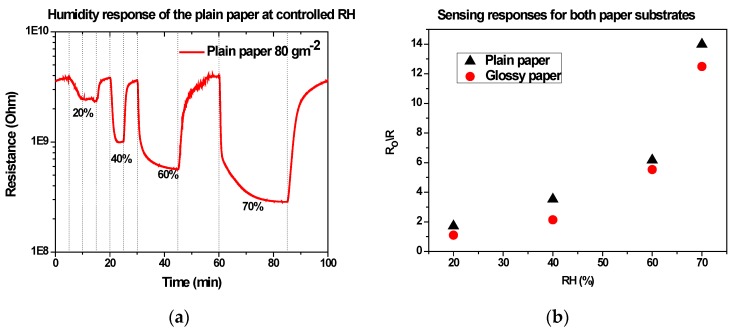
(**a**) Humidity response at controlled RH levels ranging from 20% to 70%, (**b**) sensing response for both papers as function of the RH.

Figure 5a presents the change in resistance of the uncoated 80 gm^−2^ paper-based devices under controlled RH levels ranging from 20% up to 70%, where we observe a drop in resistance as the humidity level increases. Figure 5b shows the resulting sensing response, calculated as the ratio of the electrical resistance (R_o_/R), as a function of humidity level for 80 and 200 gm^−2^ paper-based devices; the plain paper exhibits a higher response in the 20% to 50% RH rage than the glossy one.

Indeed, a porous and rough paper surface is more prone to water molecule absorption than a smooth one, since its surface area is larger. In fact, at moderate RH (40%), diffusion of water vapor further inside the volume of paper occurs through the network of air spaces among the cellulose fibers. As water progresses through the stack, an amount is sorbed onto the fibers’ surface, which results in water to lose its original concentration. The cellulose fibers absorb and desorb water into the capillary air space next to them, with little to no transmission of water molecules between them, therefore any further movement of wateroccurs almost entirely through air spaces. This absorption causes the paper resistance, as measured by the IDE electrodes, to decrease. At higher percentages of RH, water dissociation occurs on the moist cellulose fiber, creating H^+^ and OH^−^. Thus, the current can flow due to ionic conduction decreasing resistance along the IDE electrodes and resulting to a high sensing response for both papers. The dependence of electrical resistance on water diffusion through the porous structure of paper is also indicated by the difference in the response and recovery times obtained for the two paper substrates.

**Figure 6:**
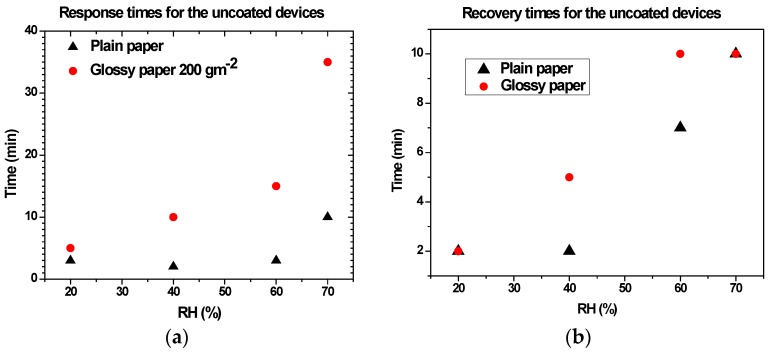
(**a**) Response time and (**b**) recovery time of the uncoated devices for both plain^2^ (triangles) and 200 glossy paper (circles).

Figure 6a shows the response times of the uncoated paper-based devices, where response time is defined as the time required for the device to reach 90% of its steady state value. We observe that the response time of the plain paper is considerably lower than that of the glossy paper. This can in part be attributed to differences in structure and porosity between the two papers due to different manufacturing processes.

Generally, a rough and porous surface is more capable of water absorption than a smooth one, which results to a faster response time. The recovery time follows the same trend as the response time with a faster dehumidification for the plain paper up to 60% RH as shown in Figure 6b. The slower recovery time at higher RH could be attributed to the presence of higher partial pressure of water vapor close to the surface of the paper substrate when it recovers through the desorption of water molecules.

The humidity sensing performance of both papers in terms of response/recovery time and sensitivity are summarized in Table 1 and are compared with previous humidity sensors made on paper substrate. We notice that porous paper even without additional functionalization exhibits a high sensitivity in a range from 0 to 70% level of humidity. Contrary to previous work found in the literature using paper substrate, in our case no additional material is used to improve sensing performances.

### 3.3. Effect of ZnO Nanoparticles on the Sensitivity of the Paper-Based Devices

The effect of humidity on paper can diminish its potential for use in electronic devices, therefore, this issue needs to be addressed. In order to eliminate the humidity effect, we propose an original solution consisting in the deposition of ZnO nanoparticles. Layers of ZnO nanoparticles were deposited using a standard and low-cost spin-coating method to allow the creation of a uniform layer on the entire surface of the paper.

**Figure 7:**
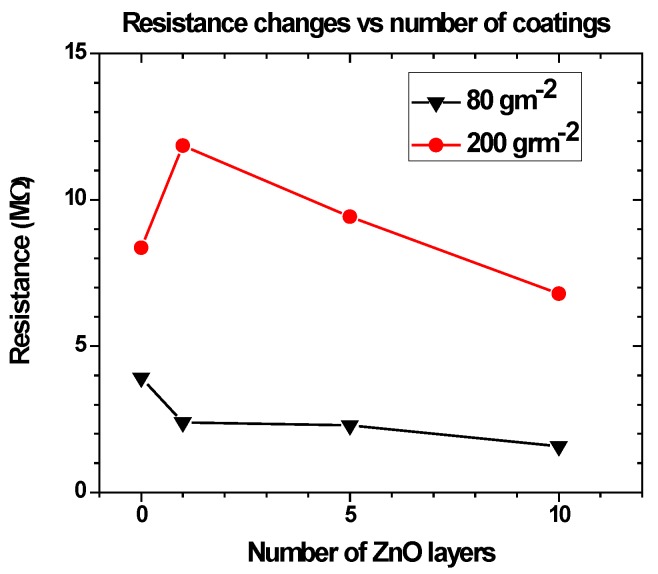
Resistance changes between the electrodes as a function of the number of the ZnO layers coated in both the 80 gm^−2^ (triangles) and the 200 gm^−2^ (circles) papers.

For both kinds of paper, resistance decreases as the number of successive spin coated layers increases. For the plain paper, a 30% reduction in resistance, from 1.96 MΩ to 1.55 MΩ, was observed after 10 deposited layers from a 40 mM sol-gel and the glossy paper exhibits a similar behavior, except for the first deposition. Upon deposition of a single ZnO layer, resistance increased compared to the uncoated paper. Subsequent layer deposition resulted in ever lower resistance, as can be seen in Figure 7. This different behavior could be attributed to different surface roughness and differences in the manufacturing process between the two papers.

**Figure 8:**
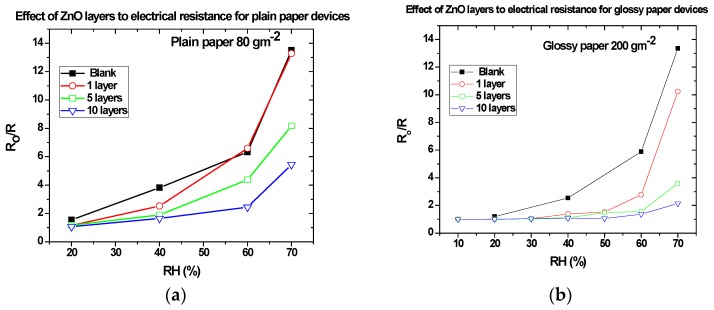
Electrical resistance changes for devices on the (**a**) 80 gm^−2^ paper coated with 1 (hollow circles), 5 (hollow squares) and 10 (hollow reverse triangles) layers of ZnO nanoparticles from a sol-gel solution of 40 mM as a function of RH and on (**b**) 200 gm^−2^ paper. Measurements in each RH were conducted in fixed time intervals of 30min.

Figure 8 shows the ratio of the electrical resistance (R_o_/R), measured between the IDE electrodes, of the 80 gm^−2^ and 200 gm^−2^ paper-based devices as a function of the RH, after each spin-coating of ZnO nanoparticles. We observe that the device’s response to humidity decreases with the number of coatings. After five deposited layers, the effect of humidity on the paper is considerably decreased. These results reveal that the ZnO layer, at least under the experimental conditions used in this work, acts as a passivation layer to humidity prohibiting water molecules from interacting with the cellulose fibers. All measurements were conducted in fixed time intervals (30 min) for each %RH, where the device’s passivation remain unchanged for the duration of each measurement.

In the case of glossy paper (Figure 8b) the decrease is more profound than that observed in the 80 gm^−2^ paper and results in the complete passivation of paper after 10 deposited layers. This can be attributed to the different morphology and fabrication process of the two different papers, as it can be seen from SEM images (Figure 4). Indeed, the ZnO nanoparticles are absorbed by the porous paper making the formation of a uniform and solid ZnO layer at the paper surface more difficult, while a smooth surface facilitates the formation of a uniform solid thin film resulting in better passivation to humidity. Even though an important factor for paper-based electronics is the effect of long-term humidity, it not considered here, since the aim of this work is to develop low-cost and disposable sensors (e.g., for agricultural applications). The long-term stability effect has been studied in an indirect way. All fabricated sensors were thoroughly tested in a period of 6 months since their fabrication time and in several cases measurements were repeated after a couple of months, with results showing only little variation. In addition, new fabricated sensors exhibited similar behavior.

The two key factors that control sensitivity towards humidity are porosity and surface area. Due to the fibrous structure of the paper, one layer of ZnO nanoparticles is not enough to create a solid thin film on the surface of the paper, so the measured sensing response is similar to that of the uncoated one. As the number of coating layers increases, more ZnO is deposited both on the surface and inside the body of the paper, causing fewer cellulose fibers to come in contact with water molecules, as most of them are blocked by the ZnO nanoparticles. The cellulose fibers gradually become completely covered, the air gaps among them are filled up and this leads to the passivation of the paper against humidity, as evidenced by measuring smaller changes in resistance.

## 4. Conclusions

We have performed a structural characterization of plain and glossy paper made bydifferent manufacturing processes in order to determine the effect of paper morphology on its humidity sensing properties. We also investigated the electrical resistance of both paper types under various humidity levels. This study showed that plain paper with porous and rough surface exhibits higher sensitivity and faster response time compared to a smooth glossy paper, proving the possibility to improve the humidity sensing performance of paper by controlling its surface porosity and roughness without additional sensing materials.

Furthermore, we demonstrated a promising method for the passivation of paper substrate towards humidity. This method consists in controlling the effect of humidity by successive spin-coated layers of ZnO nanoparticles on top of the interdigitated electrodes. Tested for both types of paper substrate, plain and glossy paper, the successive coating of the electrodes with ZnO nanoparticles induces a gradient passivation towards relative humidity changes, making the paper impervious to moisture. With this approach, total passivation of glossy paper has been achieved for relative humidity ranging from 20% to 70%. Therefore, the fabricated paper-based devices have the potential to be used for low-cost and disposable humidity sensors, and for designing selective paper-based chemical sensors insensitive to humidity. Future works will aim to use ZnO nanostructured layers for the detection of volatile organic components (VOC) like ethanol.

## Figures and Tables

**Table 1:** Humidity sensing performance of different sensor devices fabricated on paper substrate.

Reference	Year	Sensing Material	Minimum RH (%)	Response/Recovery time
[35]	2011	Polypyrrole	40	418 s/418 s
[36]	2012	Carbonnanotube	20	6 s/120 s
[37]	2014	Aluminumoxide	25	/
[38]	2015	PVA	50	/
[18]	2015	Paper	30	4 min/6 min
This work	2016	Porouspaper	20	1 min/2–10 min
This work	2016	Smooth paper	40	10 min/8 min
